# Walnut Hills Medical Society

**Published:** 1892-03

**Authors:** 


### PROCEEDINGS.

### Walnut Hills Medical Society.

Dr. Shields reported the case of a lady, thirty-five years of age, who applied to him for dental work. Before filling a cavity, he applied to it and the neighboring gum a 10 per cent. solution of cocaine. In a half hour the patient complained of weariness, and then nausea. Whisky was administered. The face became pale, the hands very cold, and soon the patient was comatose. The pupils were dilated, and, strange to say, a hazy condition of the cornea was noticed. Inhalations of nitrite of amyl were used, at intervals, for an hour, with effect, and the patient was sent home. Was called to her in the afternoon and found her in a hysterical condition, this being followed apparently by coma. Nitrite of amyl was again successfully resorted to. The haziness of the cornea was again noted.

What was the cause of this condition? Was it owing to the cocaine, or to the nervous condition superinduced? Could it have been due to contracted blood-vessels?

The speaker mentioned another case, in which the patient had a tooth removed by Dr. Hill. Two weeks later an abscess developed, and the patient consulted Dr. Shields. The third molar had been removed, and the one in front had been loosened by an abscess. He detected a sinus with the probe, cut down, found and removed a small piece of the root. The fistulous tract opened externally. It was treated by irrigation with a permanganate solution. A large scar resulted.

In a third case a decayed tooth had caused an abscess, which was lanced by a physician. Later the patient consulted Dr. Shields, who extracted the tooth and applied a lead bandage, which was tightened daily, with the result of causing the abscess to discharge internally. No scar, except that resulting from the knife.

---

Official Report.—Meeting of January 27, 1892.—The President, A. W. Johnson, M. D., in the Chair; R. C. Jones, M. D., Secretary.—Amyl Nitrite in Cocaine Nausea.—Dental Cases.

DISCUSSION.

Dr. Isham.—Have never seen any reference to the hazy condition of the cornea due to cocaine. It does induce nervous manifestations; delirium, or even coma, has occasionally followed its use. Have a limited experience with it, for the removal of small growths, opening abscesses, etc. Have usually noted some exhilaration, and occasionally mild delirium. Several years ago a homœpathic physician, in this vicinity, called at a drug store for a solution of atropia. He claimed that the druggist made a mistake, and dispensed cocaine instead of atropia. A suit for damages was threatened, but the druggist compromised for $150.

Dr. Scott.—I have not used cocaine much, but have never had any bad results from it. Is it not possible that the haziness of the cornea was due simply to the shock? Is there not always some diminution of transparency in the cornea associated with shock?

Dr. Porter.—Have had comparatively little experience with cocaine. Have used it occasionally in circumcisions and a few other minor surgical operations. Its safety and efficiency depend upon the extent to which the local circulation can be controlled. Most of the fatal accidents have occurred in operations about the rectum and genito-urinary organs, parts richly supplied with blood, and in which the circulation is not easily controlled. Cannot explain the haziness of the cornea in the case reported.

Dr. Johnstone.—Was the patient menstruating?

Dr. Shields.—I do not know, as I never ask such questions, fearing offense.

Dr. Johnstone.—How much of a role does tuberculosis play in the destruction of teeth?

Dr. Shields.—I have noticed nothing of its effects.

